# A retrospective cohort study of stroke onset: implications for characterizing short term effects from ambient air pollution

**DOI:** 10.1186/1476-069X-10-87

**Published:** 2011-10-06

**Authors:** Julie YM Johnson, Paul J Villeneuve, Dion Pasichnyk, Brian H Rowe

**Affiliations:** 1 Population Studies Division, Health Canada, Ottawa, Ontario, Canada, 50 Columbine Driveway, Tunney's Pasture, Room 165, PL0801A Ottawa, Ontario K1A 0K9, Canada; 2 Division of Occupation and Environmental Health, Dalla Lana School of Public Health, University of Toronto, Toronto, Ontario, Canada, 155 College Street, Health Science Building, 6th floor, Toronto, Ontario, M5T 3M7, Canada; 3 Department of Emergency Medicine, Faculty of Medicine and Dentistry and School of Public Health, University of Alberta, Edmonton, Alberta, Canada, 2J2.00 WC Mackenzie Health Sciences Centre, Edmonton, Alberta, T6G 2R7, Canada

**Keywords:** Air pollution, stroke, survey, emergency service

## Abstract

**Background:**

Case-crossover studies used to investigate associations between an environmental exposure and an acute health response, such as stroke, will often use the day an individual presents to an emergency department (ED) or is admitted to hospital to infer when the stroke occurred. Similarly, they will use patient's place of residence to assign exposure. The validity of using these two data elements, typically extracted from administrative databases or patient charts, to define the time of stroke onset and to assign exposure are critical in this field of research as air pollutant concentrations are temporally and spatially variable. Our a priori hypotheses were that date of presentation differs from the date of stroke onset for a substantial number of patients, and that assigning exposure to ambient pollution using place of residence introduces an important source of exposure measurement error. The objective of this study was to improve our understanding on how these sources of errors influence risk estimates derived using a case-crossover study design.

**Methods:**

We sought to collect survey data from stroke patients presenting to hospital EDs in Edmonton, Canada on the date, time, location and nature of activities at onset of stroke symptoms. The daily mean ambient concentrations of NO_2 _and PM_2.5 _on the self-reported day of stroke onset was estimated from continuous fixed-site monitoring stations.

**Results:**

Of the 336 participating patients, 241 were able to recall when their stroke started and 72.6% (95% confidence interval [CI]: 66.9 - 78.3%) experienced stroke onset the same day they presented to the ED. For subjects whose day of stroke onset differed from the day of presentation to the ED, this difference ranged from 1 to 12 days (mean = 1.8; median = 1). In these subjects, there were no systematic differences in assigned pollution levels for either NO_2 _or PM_2.5 _when day of presentation rather than day of stroke onset was used. At the time of stroke onset, 89.9% (95% CI: 86.6 - 93.1%) reported that they were inside, while 84.5% (95% CI: 80.6 - 88.4%) reported that for most of the day they were within a 15 minute drive from home. We estimated that due to the mis-specification of the day of stroke onset, the risk of hospitalization for stroke would be understated by 15% and 20%, for NO_2 _and PM_2.5_, respectively.

**Conclusions:**

Our data suggest that day of presentation and residential location data obtained from administrative records reasonably captures the time and location of stroke onset for most patients. Under these conditions, any associated errors are unlikely to be an important source of bias when estimating air pollution risks in this population.

## Background

Over the past decade, several epidemiological studies have demonstrated that short-term (i.e., within days) fluctuations in ambient air pollution can increase in the risk of stroke [1-4]. These studies have typically employed either a time-series or case-crossover design to describe associations between the daily number of stroke events and air pollution levels. A variety of measures have been used to identify stroke events including: presentation to emergency departments (EDs) [2,4], admittance to hospital [1,5-7], and death [8,9]. Regardless of the manner in which the stroke outcome is defined, the calendar dates associated with these events, typically extracted from hospital administrative data, has been used to define the time (i.e., day) of stroke onset. However, there may be considerable delays from stroke onset to when an individual presents to hospital or dies that may distort the risk estimates from these studies. Lokken et al [10] found that the onset of stroke symptoms frequently occurred more than one full calendar day before hospital admission, and that the impact from this misclassification of the timing of stroke onset produces air pollution risk estimates that may be understated by as much as 40% [10]. Apart from that study, we know of no other research that has evaluated potential biases that may arise from inaccuracies related to the timing of the onset of stroke.

Another fundamental assumption of the time-series or case-crossover study design is that exposure can be adequately characterized using ambient concentrations from fixed-site monitoring networks. Some stroke studies have regressed the daily number of stroke events against a city-wide measure of ambient pollution [4,8], while in others, exposure has been assigned at a finer spatial scale by incorporating information on place of residence data [6,10]. However, such an approach does not necessarily reduce exposure measurement misclassification because the individual may have been away from home or outside the city at the time of stroke onset. Furthermore, for some pollutants, ambient concentrations at the patient's place of residence may not accurately reflect the salient exposure because they were inside when there stroke occurred. Recently, a study of models of traffic-related pollution estimates that included time-activity patterns suggested that the difference between the mobility-based model and residence-only models may lead to risk estimates biased toward the null [11]. Investigations into the effect of misclassification of ambient air pollution exposure on time-series models of acute health outcomes have shown that the Berkson type error due to differences between individual exposures and city-wide average exposures has a relatively modest influence on observed risk estimates [12,13].

The objective of this study was to examine the extent of misclassification of exposure based on data on patient residence and hospital admission time and date to increase our understanding of the possible biases in ambient air pollution risk estimates arising from the use of administrative datasets. We previously reported positive associations between ambient measures of air pollution and ED stroke visits among residents in the city of Edmonton, Canada [4]. Here, we compared responses from a survey of stroke and transient ischemic attack (TIA) patients presenting to two Edmonton area hospitals, with individual data on patient location at stroke and patient-reported time of stroke onset, and compared their responses to similar characteristics collected from administrative records.

## Methods

### Stroke patient data

The setting for this study was Edmonton, Alberta, Canada. The two study sites were the University of Alberta Hospital (UAH), an academic teaching hospital with designated stroke and neuroscience units, and the Royal Alexandra Hospital, a high-volume clinical teaching hospital with neurosurgical and neurological consultants available. Both sites are regional trauma centers, are staffed by full-time certified emergency physicians, and are involved in learner training. Given the presence of the stroke service at the UAH, more acute stroke patients present to this setting than other hospitals within the region.

Eligible patients were those who presented to the ED of the study sites between June 10, 2009 and May 19, 2010, and who had a discharge diagnosis of hemorrhagic or ischemic stroke or TIA: International Classification of Diseases, 10^th ^revision (ICD-10) codes I60 through I64, I67, and G45. Data were collected on excluded patients: reason for exclusion, triage date, and stroke classification.

At least one half-hour after examination and no more than 24 hours after presenting to hospital, and following the confirmation of a diagnosis of stroke or TIA by the attending physician, patients or their families were approached by a trained research assistant to participate in the study. Following informed written consent, the research assistant administered a questionnaire that collected information on the patient's place of residence, cigarette smoking behavior, and medical history. Patients were also asked to recall the date and time of stroke onset (i.e., when they first experienced stroke symptoms), where they were at onset (inside, outside, in vehicle), and what activity they were performing (resting, routine daily activity, exercise). They were also asked to indicate where they had spent most of their time on the day of stroke onset with one of the following responses: a) "Most of the time I was at home or within a 15 minute drive from my home", b) "Most of the time I was more than a 15 minute drive from home, but still in the Edmonton area", or c) "Most of the time I was outside of the Edmonton area".

Following the discharge, admission/transfer, or death, additional data for each patient were obtained from medical charts. Data extracted from the medical charts included: sex, age, postal code, ICD-10 code, outcome (e.g., admission, discharge, death), triage time and date, time and date of stroke onset, presence of heart disease, history of stroke, use of anti-hypertensive drugs and insulin or oral hypoglycemic medication (used as indicators of the presence of hypertension and type 2 diabetes, respectively), and the Canadian Triage and Acuity Scale (CTAS) score. The CTAS is an assessment tool for prioritizing ED patients upon arrival based on presenting complaints and classifies patients into five categories: resuscitation, emergent, urgent, less urgent and non-serious complaint [14].

### Ambient Air Pollution Exposure

Hourly concentrations of ambient NO_2 _(ppb) and PM_2.5 _(particulate matter < 2.5 microns in diameter) (μg/m^3^) were obtained from 3 continuous monitoring stations within the city of Edmonton during the study period. The monitors are part of the National Air Pollution Surveillance Network maintained by Environment Canada [15]. Daily mean values were calculated by taking the average of measures obtained from the three monitoring stations for the period January 01, 2009 to May 19, 2010.

### Statistical and Data Analyses

We identified those patients whose self-reported date of stroke onset from the survey differed from the date of presentation to the ED captured by the medical chart. Chi-squared tests were applied to evaluate differences in patient characteristics between these patients and those whose reported day of stroke onset occurred on the day of presentation. Similar analyses were also undertaken to evaluate differences in patient characteristics among those who were close to home on the day of their stroke to those who were further away. Characteristics that were evaluated in these analyses included: age-group, sex, previous stroke, CTAS, season, smoking status, and history of hypertension, diabetes, and heart disease.

We also evaluated the effect of misclassification of the timing of stroke onset on assigned ambient concentrations of PM_2.5 _and NO_2_. This was performed for those subjects whose self-reported day of stroke onset differed from the day of presentation to the ED. For each of these subjects, we calculated the difference in daily city-wide mean NO_2 _and PM_2.5 _levels between these two days. Histograms were then created to describe the frequency distribution of these differences, and a normal distribution was then fit to this distribution. In addition, we estimated the bias resulting from the mis-specification of stroke onset utilizing a previously developed formula [10]. Specifically, attenuation factor for the risk estimate was estimated by c = var(x)/{var(x) + var(x* - x)}; where × represents the exposure at the time of stroke onset, and x* represents the exposure on the mis-specified date of onset.

### Ethics

Ethics approval for this study was provided by the Health Research Ethics Board (Biomedical Panel) at the University of Alberta and by Health Canada's Research Ethics Board.

## Results

### Recruitment

From 760 stroke patients presenting during the study period, 239 were ineligible to participate because they resided outside of the metropolitan area of Edmonton (n = 164) or had discharge diagnoses that were incompatible with the study criteria (n = 75). Eight of the 521 eligible participants were unable to participate due to language barrier and 4 died in ED or were terminal. Ninety-five were missed (left the hospital or were transferred before a research assistant was able to administer the questionnaire), 64 were unable to respond, and 14 refused to participate (2.7% of 521).

Non-participants differed somewhat from study participants. Compared to the distribution of stroke sub-types among study participants, the distribution among those unable to respond at time of interview was shifted away from the TIA stroke classification and towards ischemic and hemorrhagic stroke (χ^2 ^= 14.0, *p *= 0.001). The distribution of sub-types among those who were missed by research assistants at the time of interview was not different compared to participants (χ^2 ^= 1.94, *p *= 0.38).

### Sample

A total of 336 patients participated in the survey (173 men, 163 women) (Table 1). Ischemic stroke was the most common type, accounting for more than two-thirds of all cases. Almost two thirds of all patients who participated in the survey experienced a stroke for the first time. The prevalence of hypertension and diabetes in our survey population was 71.9% (95% CI: 67.1 - 76.8%) and 21.5% (95% CI: 17.1 - 25.9%), respectively. Most patients reported being within a 15 minute drive from home on the day when stroke onset symptoms occurred (84.5%; 95% CI: 80.6 - 88.4%) and being inside at the time of stroke onset (89.9%; 95% CI: 86.6 - 92.1%).

**Table 1:** Characteristics of 337 stroke patients who participated in the survey

Characteristic	Category	N	%
Sex*	Male	173	51.5

	Female	163	48.5

Age *	< 65 years	105	31.3

	65-74 years	67	19.9

	75-84 years	88	26.2

	≥ 85 years	76	22.6

Stroke sub-type *	TIA	67	19.9

	Ischemic	226	67.3

	Hemorrhagic	41	12.2

	Unknown	2	0.6

Previous stroke *	Yes	114	34.0

	No	221	66.0

Hypertension *	Yes	241	71.9

	No	94	28.1

Diabetes *	Yes	72	21.5

	No	263	78.5

Smoking status †	Current regular	32	9.5

	Current occasional	53	15.8

	Former	126	37.5

	Never	122	36.3

	Unknown	3	0.9

Location at stroke †	≤ 15 minute drive from home	283	84.5

	> 15 minute drive from home, but in Edmonton	47	14.0

	Outside of Edmonton	5	1.5

Timing of stroke onset †	Morning (06:00-11:59)	100	29.8

	Afternoon (12:00 - 17:59)	70	20.8

	Evening (18:00 - 23:59)	44	13.1

	Overnight (00:00 - 05:59)	19	5.7

	Unknown	103	30.7

Awake at stroke †	Yes	248	73.8

	No	51	15.2

	Unknown	37	11.0

Activity at stroke †	Sleeping	79	23.5

	Exercising	15	4.5

	Sitting/resting	167	49.7

	Exerting at work	11	3.3

	Other	64	19.0

Inside/outside at stroke †	Inside	302	89.9

	Outside	15	4.5

	In vehicle	16	4.8

	Other	3	0.9

* Data from medical chart data

† Data from survey data

### Presenting Times

Of the 336 participants, 241 (71.7%; 95% CI: 66.9 - 76.6%) were able to recall the day on which symptoms of their stroke started. For 27.4% (95% CI: 21.7 - 33.1%) of these 241 patients the date of self-reported stroke onset was at least one day before the date of presentation to ED. The number of days between the onset of stroke and presentation to ED ranged from 1 to 12 days (median = 1.0, mean = 1.8, 95% CI: 1.4 - 2.3 days). Those presenting with higher acuity conditions were more likely to present to ED on the same day they first experienced stroke symptoms (χ^2 ^= 14.7, p < 0.001); however, stroke sub-type was not associated with delay (χ^2 ^= 2.35, p = 0.31) (Table 2). The difference in hours between time of onset and time of presentation for 231 patients who could recall the time of stroke onset ranged from 0 to 191 hr (median = 3 hr, mean = 11.9 hr, 95% CI: 8.7 - 15.0 hrs); 59.7% (95% CI: 53.4 - 66.1%) presented to ED within 3 hours of onset.

**Table 2:** Selected characteristics for stroke patients whose self-reported day of stroke onset was the same as the day they presented to an ED compared to those that differed

	Stroke onset = DOP (N = 175)		Stroke onset ≠ DOP (N = 66)		*p*
**Characteristic**	**n**	**%**	**n**	**%**	

Age					

< 65 years	49	28.0	27	40.9	0.20

65-74 years	38	21.7	14	21.2	

75-84 years	50	28.6	12	18.2	

≥ 85 years	38	21.7	13	19.7	

Sex					

Male	88	50.3	38	57.6	0.31

Female	87	49.7	28	42.4	

Previous stroke					

Yes	67	38.5	17	25.8	0.06

No	107	61.5	49	74.2	

Stroke subtype					

TIA	41	23.6	11	16.7	0.31

Ischemic	111	63.8	49	74.2	

Hemorrhagic	22	12.6	6	9.1	

CTAS					

Emergent	111	63.4	24	36.4	0.001

Urgent	61	34.9	41	62.1	

Less urgent	3	1.7	1	1.5	

Hypertension					

Yes	124	71.3	50	75.8	0.49

No	50	28.7	16	24.2	

Diabetes					

Yes	35	20.1	15	22.7	0.66

No	139	79.9	51	77.3	

Heart disease					

Yes	56	32.2	21	31.8	0.96

No	118	67.8	45	68.2	

Smoking status					

≥ 15 cigarettes/day	14	8.1	5	7.6	0.52

< 15 cigarettes/day	24	13.9	14	21.2	

Former	71	41.0	27	40.9	

Never	64	37.0	20	30.3	

DOP = Day of presentation to the ED

### Associations with Ambient Air Pollution

**Figure 1:**
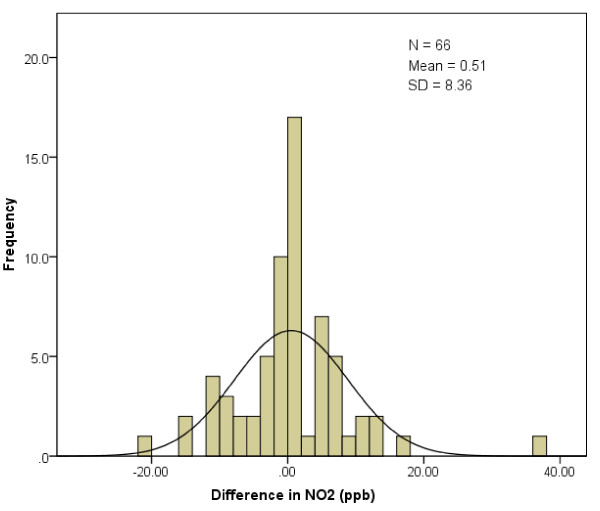
Frequency distribution of differences* in assigned ambient NO_2 _concentrations among 66 stroke patients whose self-reported day of stroke onset differed from the day of presentation. * The difference was calculated by subtracting the daily mean ambient NO_2 _concentration on the self-reported day of stroke onset from the daily mean concentration on the day the patient presented to the ED.

**Figure 2:**
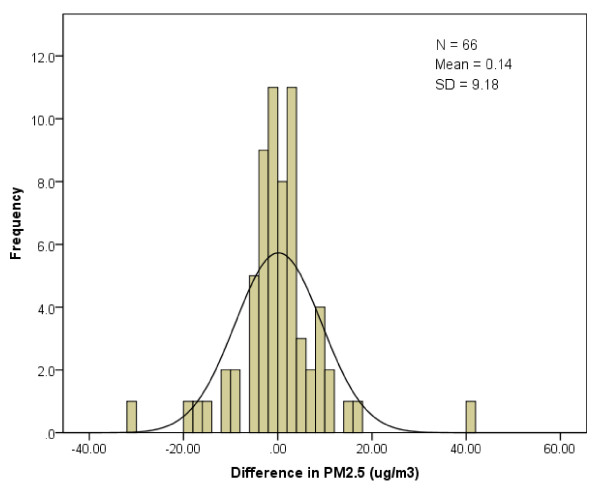
Frequency distribution of differences* in assigned ambient PM_2.5 _concentrations among 66 stroke patients whose self-reported day of stroke onset differed from the day of presentation. * The difference was calculated by subtracting the daily mean ambient PM_2.5 _concentration on the self-reported day of stroke onset from the daily mean concentration on the day the patient presented to the ED.

For patients who did not present to the ED until at least one day after the onset of stroke symptoms, the mean difference in city-wide mean NO_2 _between the day of stroke onset and the day of presentation to ED was only 0.51 ppb (95% CI: -1.55 - 2.56 ppb, Figure 1). We repeated these calculations for PM_2.5_, for which the mean difference was 0.14 μg/m^3 ^(95% CI: -2.11 - 2.40 μg/m^3^, Figure 2). The differences of NO_2 _or PM_2.5 _were not normally distributed (Figures 1 and 2). Based on the variances of exposure at onset and at ED presentation, the approximated reduction in stroke risk using the exposure data for patients with delays would be 16% for a linear regression model of NO_2 _effect, and 20% for a model of PM_2.5 _effect.

Comparisons of patient characteristics for those who were most often within a 15 minute drive from home versus those who were farther away on the day of their stroke are presented in Table 3. Those who were younger (χ^2 ^= 25.4, p < 0.001) and hypertensive (χ^2 ^= 12.1, p = 0.001) were more likely to be further away from home at the time of stroke onset. No statistically significant differences were found between those within a 15 minute drive and those who were further away for cigarette smoking status, season, or sub-type of stroke.

**Table 3:** Selected characteristics of stroke patients who were most often more than a 15 minute drive away from home on the day of stroke onset compared to those who were nearer to home

	< 15 minute drive (N = 283)		≥ 15 minute drive (N = 52)		*P*
**Characteristic**	**n**	**%**	**n**	**%**	

Age					

< 65 years	74	26.1	31	59.6	< 0.001

65-74 years	57	20.1	9	17.3	

75-84 years	84	29.7	4	7.7	

≥ 85 years	68	24.0	8	15.4	

Sex					

Male	139	49.2	33	63.5	0.06

Female	144	50.8	19	36.5	

Previous stroke					

Yes	98	35.0	16	30.8	0.58

No	184	65.0	36	69.2	

Stroke subtype					

TIA	51	18.1	16	30.8	0.11

Ischemic	194	69.0	31	59.6	

Hemorrhagic	36	12.8	5	9.6	

CTAS					

Resuscitation	3	1.1	0	0.0	0.84

Emergent	148	52.3	27	51.9	

Urgent	129	45.6	24	46.2	

Less urgent	3	1.1	1	2.0	

Hypertension					

Yes	213	75.5	27	51.4	0.001

No	69	24.5	25	48.1	

Diabetes					

Yes	62	22.0	10	19.2	0.66

No	220	78.0	42	80.8	

Heart disease					

Yes	90	31.9	10	19.2	0.07

No	192	68.1	42	80.8	

Smoking status					

≥ 15 cigarettes/day	26	9.3	6	11.5	0.16

< 15 cigarettes/day	41	14.6	12	23.1	

Former	113	40.3	13	25.0	

Never	100	35.7	21	40.4	

DOP = Day of presentation to the ED

## Discussion

Recording stroke onset time and minimizing delay between onset and presentation to ED are critical for patients with cerebral infarction because effective treatment with recombinant tissue-type plasminogen activator has a post-onset treatment window of 4.5 hours [16]. Documentation of onset time in the stroke patient population is often missing or inaccurate. For example, examinations of the standards of stroke care in the Paul Coverdell National Stroke Surveillance database revealed that time of stroke onset was missing from 58% of 56,969 stroke cases in the US from 2005 to 2007 [17]. Possible patient factors contributing to missing such data include lack of patient recognition of stroke symptoms, symptom onset during sleep, cognitive impairment upon presentation, and lack of preliminary evidence of cerebral infarction. Possible physician factors contributing to missing or inaccurate data include lack of physician recognition of the importance of documenting stroke onset, busy/chaotic ED environment, patients' inability to recall events, presentation within/outside window driving clinical action, and lack of clear evidence of diagnosis at the time of charting.

While missing onset data can have serious clinical implications in stroke management, our results suggest that using date and time of presentation to ED from an administrative database as a surrogate for date of stroke onset should not lead to a strong exposure misclassification effect on risk estimates obtained from case-crossover studies. Time series and case-crossover analyses are effective study designs to describe associations between short-term fluctuations in ambient air pollution measures and adverse health outcomes. They may be, nonetheless, vulnerable to important sources of bias. We evaluated the potential bias from misclassifying the time of disease onset using administrative hospitalization data, and the potential for exposure misclassification by exploring activity patterns among stroke patients at the time of stroke onset. A systematic difference in ambient NO_2 _and PM_2.5 _levels could not be detected between day of stroke onset and day of presentation to hospital. Specifically, the mean difference was close to zero.

Our study follows up on a previous investigation that reported misclassification effects due to incomplete or inaccurate data on day of the outcome when dependent on hospital admission databases [10]. Comparing PM_2.5 _concentrations at admission and at onset observed among 1,101 stroke patients in Boston, Lokken et al [10] reported a mean difference of -0.1 μg/m^3^. In our data, the difference in concentration between time of presentation and onset was similar (0.14 μg/m^3^). The Boston study approximated a 50% reduction in ischemic stroke risk based on variance of PM_2.5 _concentrations at onset and admission times under a linear regression model. The same approximation method applied to our PM_2.5 _data showed a reduction of 20%, suggesting less variance in exposure misclassification in our Edmonton data. Employing the 16% attenuation factor for misclassification of NO_2 _calculated from the present data, to the regression coefficient for an interquartile increase in the 3 day average NO_2 _we reported in our earlier case-crossover study of ambient air pollution effects on admissions to Edmonton EDs for stroke [4], the observed odds ratio of 1.26 would have been reduced from 1.32.

Several systematic differences between the Boston study [10] and ours should be noted. Delays to ED presentation may be greater among a US population than a Canadian one, for whom accessibility and universality of hospital ED care reduces the potential for individuals of lower socioeconomic levels to postpone seeking medical treatment, as they do in the US [18]. Secondly, in the Boston study an onset time estimation method used a 6-hour time window when the onset time could not be given within a 15-minute time window; thus, onset date data was known or estimated for nearly 100% of patients. Imputing stroke onset back in time within a 6-hour window can increase the likelihood of being classified as delayed. In contrast, we did not use an onset estimation method and had stroke onset data for roughly 72% of patients (similar to findings by others [19]). Finally, the Boston study did not include hemorrhagic strokes or TIAs. Since we observed no association between delays and subtype, this likely did not contribute to the differences in results between the studies. Nevertheless, there was an increased likelihood of a greater effect of misclassification in the Boston study data because the proportion of patients classified as delayed at least one day was higher (53%) and was well in excess of the range reported here and by others (18 - 27%) [20,21]. In the Boston study, Lokken et al also ran case-crossover models on simulated datasets reflecting the distribution of delays [10]. Logistic regression β parameters from models using stroke onset time data were 60 to 66% less than those using hospital admission data. It is unclear if we also would have found a systematic reduction in the short term effect of ambient NO_2 _or PM_2.5 _on stroke if onset date was used instead of ED presentation date in a case-crossover model.

We found that higher CTAS scores were strongly associated with less delay in presentation to hospital after stroke symptoms first appeared. Our results echo those from an examination of delay risk factors including the modified National Institute of Health Stroke Scale (nMIHSS), a measure neurological impairment [19]. Those with high nMIHSS scores had less delay to arrival and, as in our study, previous stroke and stroke sub-type were not associated with delay. Similar to our findings with respect to presentation dates, others found that delay to hospital admission was not associated with age, smoking, hypertension, diabetes, or heart disease, and that those with previous stroke are marginally less likely to be delayed [21].

A high response rate was a strength of this study (2.7% refused to participate); however, there were many stroke patients who were missed or unable to respond. The data available for non-participants showed that the proportion of patients who suffered more clinically severe stroke events (ischemic and hemorrhagic stroke) was higher among those unable to respond than it was among participants. Nevertheless, given that sub-type was not associated with delay to ED, there is no indication that the sub-type distribution among non-participants would have been a source of bias of our results.

Our data revealed that a very high proportion of stroke patients were indoors during stroke onset, which is not surprising, as adult Canadians spend more than ¾ of their time indoors, even in summer months [22]. Personal exposure to NO_2 _and PM_2.5 _can be different than background exposure measurements, and in Canada the difference can be dependent on season, the use of gas heating, gas stoves, and air conditioning [23-25]. For these reasons, data on, or models of indoor air pollution exposure could substantially improve our understanding of the effect of personal exposure to indoor air pollution on acute health outcomes in Canada.

Spatial variability is an additional source of exposure measurement error that may have affected our results. These errors are caused by the inability of centralized monitoring station data to precisely reflect ambient levels for individuals. In our study we used city-wide daily averages; our measurements were, thus, affected by Berkson-type error [12]. Arguing that measurement error in air pollution data is often distributed lognormally, Goldman et al concluded that spatial error can result in inflation of a risk ratio on a per unit concentration basis, depending on the degree of spatial variability present in the study area [26]. This is a critical source of error, then, that could have a stronger effect on risk estimates than would the error with respect to timing of stroke onset inherent in hospital administrative databases we observed.

## Conclusions

Our results demonstrated that a non-negligible proportion of stroke patients presented to ED after a day or more following onset of symptoms. Despite this observation, for those who were delayed, the differences in ambient NO_2 _and PM_2.5 _between onset and triage dates were not significantly different from zero; thus, the potential impact of exposure misclassification due to the use of ED presentation time on case-crossover risk estimates of pollution effects on stroke events is likely to be small.

## List of abbreviations

CI: confidence interval; CTAS: Canadian Triage and Acuity Scale; ED: emergency department; ICD-10: International Classification of Diseases, 10^th ^revision; NO_2_: nitrogen dioxide; PM_2.5_: particulate matter < 2.5 microns in diameter; TIA: transient ischemic attack.

## Competing interests declaration

The authors declare that they have no competing interests.

## Authors' contributions

JYMJ participated in study design, conducted analyses, and drafted and revised the manuscript. PJV originated study concept, participated in study design, advised analyses, and revised the manuscript. DP collected data and participated in drafting the manuscript. BHR participated in the study design, supervised data collection, and revised the manuscript. All authors read and approved the final manuscript.

## References

[B1] Chan CC Chuang KJ Chien LC Chen WJ Chang WT Urban air pollution and emergency admissions for cerebrovascular diseases in Taipei, Taiwan Eur Heart J 2006 27 1238 1244 16537554 10.1093/eurheartj/ehi835

[B2] Jalaludin B Morgan G Lincoln D Sheppeard V Simpson R Corbett S Associations between ambient air pollution and daily emergency department attendances for cardiovascular disease in the elderly (65+ years), Sydney, Australia J Expo Sci Environ Epidemiol 2006 16 225 237 10.1038/sj.jea.7500451 16118657

[B3] O'Donnell MJ Fang J Mittleman MA Kapral MK Wellenius GA Fine particulate air pollution (PM2.5) and the risk of acute ischemic stroke Epidemiology 2011 22 422 431 10.1097/EDE.0b013e3182126580 21399501 PMC3102528

[B4] Villeneuve PJ Chen L Stieb D Rowe BH Associations between outdoor air pollution and emergency department visits for stroke in Edmonton, Canada Eur J Epidemiol 2006 21 689 700 10.1007/s10654-006-9050-9 17048082

[B5] Low RB Bielory L Qureshi AI Dunn V Stuhlmiller DF Dickey DA The relation of stroke admissions to recent weather, airborne allergens, air pollution, seasons, upper respiratory infections, and asthma incidence, September 11, 2001, and day of the week Stroke 2006 37 951 957 10.1161/01.STR.0000214681.94680.66 16527994

[B6] Oudin A Stroh E Stromberg U Jakobsson K Bjork J Long-term exposure to air pollution and hospital admissions for ischemic stroke. A register-based case-control study using modelled NO(x) as exposure proxy BMC Public Health 2009 9 301 10.1186/1471-2458-9-301 19691845 PMC2736944

[B7] Wang XY Barnett AG Hu W Tong S Temperature variation and emergency hospital admissions for stroke in Brisbane, Australia, 1996-2005 Int J Biometeorol 2009 53 535 541 10.1007/s00484-009-0241-4 19506912

[B8] Yorifuji T Kawachi I Sakamoto T Doi H Associations of outdoor air pollution with hemorrhagic stroke mortality J Occup Environ Med 2011 53 124 126 10.1097/JOM.0b013e3182099175 21270652

[B9] Vidale S Bonanomi A Guidotti M Arnaboldi M Sterzi R Air pollution positively correlates with daily stroke admission and in hospital mortality: a study in the urban area of Como, Italy Neurol Sci 2010 31 179 182 20119741 10.1007/s10072-009-0206-8

[B10] Lokken RP Wellenius GA Coull BA Burger MR Schlaug G Suh HH Mittleman MA Air pollution and risk of stroke: underestimation of effect due to misclassification of time of event onset Epidemiology 2009 20 137 142 10.1097/EDE.0b013e31818ef34a 19244659 PMC2888684

[B11] Setton E Marshall JD Brauer M Lundquist KR Hystad P Keller P Cloutier-Fisher D The impact of daily mobility on exposure to traffic-related air pollution and health effects estimates J Expo Sci Environ Epidemiol 2011 21 42 48 10.1038/jes.2010.14 20588325

[B12] Zeger SL Thomas D Dominici F Samet JM Schwartz J Dockery D Cohen A Exposure measurement error in time-series studies in air pollution: Concepts and consequences Environ Health Perspect 2000 108 419 426 10.1289/ehp.00108419 10811568 PMC1638034

[B13] Sheppard L Slaughter JC Schildcrout J Liu LJS Lumley T Exposure and measurement contributions to estimates of acute air pollution effects J Expo Anal Environ Epidemiol 2005 15 366 376 10.1038/sj.jea.7500413 15602584

[B14] Murray M Bullard M Grafstein E Revisions to the Canadian Emergency Department Triage and Acuity Scale implementation guidelines CJEM 2004 6 421 427 17378961

[B15] National Air Pollution Surveillance Program (NAPS) http://www.ec.gc.ca/rnspa-naps/default.asp?lang=En&n=5C0D33CF-1

[B16] Shobha N Buchan AM Hill MD Thrombolysis at 3-4.5 hours after acute ischemic stroke onset--evidence from the Canadian Alteplase for Stroke Effectiveness Study (CASES) registry Cerebrovasc Dis 2011 31 223 228 10.1159/000321893 21178345

[B17] George MG Tong X McGruder H Yoon P Rosamond W Winquist A Hinchey J Wall HK Pandey DK Paul Coverdell National Acute Stroke Registry Surveillance - four states, 2005-2007 MMWR Surveill Summ 2009 58 1 23 19893482

[B18] Krajewski SA Hameed SM Smink DS Rogers SO Access to emergency operative care: A comparative study between the Canadian and American health care systems Surgery 2009 146 300 307 10.1016/j.surg.2009.04.005 19628089

[B19] Maestroni A Mandelli C Manganaro D Zecca B Rossi P Monzani V Torgano G Factors influencing delay in presentation for acute stroke in an emergency department in Milan, Italy Emerg Med J 2008 25 340 345 10.1136/emj.2007.048389 18499815

[B20] Lacy CR Suh DC Bueno M Kostis JB Delay in presentation and evaluation for acute stroke: Stroke Time Registry for Outcomes Knowledge and Epidemiology (S.T.R.O.K.E.) Stroke 2001 32 63 69 10.1161/01.STR.32.1.63 11136916

[B21] Barr J McKinley S O'Brien E Herkes G Patient recognition of and response to symptoms of TIA or stroke Neuroepidemiology 2006 26 168 175 10.1159/000091659 16493205

[B22] Leech JA Nelson WC Burnett RT Aaron S Raizenne ME It's about time: a comparison of Canadian and American time-activity patterns J Expo Anal Environ Epidemiol 2002 12 427 432 10.1038/sj.jea.7500244 12415491

[B23] Clark NA Allen RW Hystad P Wallace L Dell SD Foty R Dabek-Zlotorzynska E Evans G Wheeler AJ Exploring variation and predictors of residential fine particulate matter infiltration Int J Environ Res Public Health 2010 7 3211 3224 10.3390/ijerph7083211 20948956 PMC2954577

[B24] Gilbert NL Gauvin D Guay M Heroux ME Dupuis G Legris M Chan CC Dietz RN Levesque B Housing characteristics and indoor concentrations of nitrogen dioxide and formaldehyde in Quebec City, Canada Environ Res 2006 102 1 8 10.1016/j.envres.2006.02.007 16620807

[B25] Sorensen M Loft S Andersen HV Raaschou-Nielsen O Skovgaard LT Knudsen LE Nielsen IV Hertel O Personal exposure to PM2.5, black smoke and NO2 in Copenhagen: relationship to bedroom and outdoor concentrations covering seasonal variation J Expo Anal Environ Epidemiol 2005 15 413 422 10.1038/sj.jea.7500419 15674319

[B26] Goldman GT Mulholland JA Russell AG Strickland MJ Klein M Waller LA Tolbert PE Impact of exposure measurement error in air pollution epidemiology: Effect of error type in time-series studies Environ Health 2011 10 61 10.1186/1476-069X-10-61 21696612 PMC3146396

